# Ofd1 Controls Dorso-Ventral Patterning and Axoneme Elongation during Embryonic Brain Development

**DOI:** 10.1371/journal.pone.0052937

**Published:** 2012-12-27

**Authors:** Anna D'Angelo, Amalia De Angelis, Bice Avallone, Immacolata Piscopo, Roberta Tammaro, Michèle Studer, Brunella Franco

**Affiliations:** 1 Telethon Institute of Genetics and Medicine (TIGEM), Via Pietro Castellino 111, Naples, Italy; 2 Department of Biological Science, University of Naples “Federico II”, Naples, Italy; 3 Medical Genetics, Department of Pediatrics, Federico II University, Naples, Italy; Indiana University School of Medicine, United States of America

## Abstract

Oral-facial-digital type I syndrome (OFDI) is a human X-linked dominant-male-lethal developmental disorder caused by mutations in the *OFD1* gene. Similar to other inherited disorders associated to ciliary dysfunction OFD type I patients display neurological abnormalities. We characterized the neuronal phenotype that results from *Ofd1* inactivation in early phases of mouse embryonic development and at post-natal stages. We determined that Ofd1 plays a crucial role in forebrain development, and in particular, in the control of dorso-ventral patterning and early corticogenesis. We observed abnormal activation of Sonic hedgehog (Shh), a major pathway modulating brain development. Ultrastructural studies demonstrated that early *Ofd1* inactivation results in the absence of ciliary axonemes despite the presence of mature basal bodies that are correctly orientated and docked. *Ofd1* inducible-mediated inactivation at birth does not affect ciliogenesis in the cortex, suggesting a developmental stage-dependent role for a basal body protein in ciliogenesis. Moreover, we showed defects in cytoskeletal organization and apical-basal polarity in *Ofd1* mutant embryos, most likely due to lack of ciliary axonemes. Thus, the present study identifies *Ofd1* as a developmental disease gene that is critical for forebrain development and ciliogenesis in embryonic life, and indicates that Ofd1 functions after docking and before elaboration of the axoneme *in vivo*.

## Introduction

The forebrain is the most anterior part of the central nervous system (CNS) and derives from a simple layer of neuroepithelial cells, which becomes specified along the antero-posterior (AP) and dorsal-ventral (DV) axes by the action of various signalling molecules such as Sonic hedgehog (Shh) and Wnt family members [1].

Recently, numerous studies have revealed that the primary cilium plays a crucial role in modulating critical signalling pathways during CNS development [2], [3], [4], [5], [6], [7], [8], [9]. Primary cilia are single organelles present on almost all mammalian cells and composed of a basal body and an axoneme of 9 couples of microtubules. They are essential for the transduction of various signalling pathways controlled by Shh, Wnt and Planar Cell Polarity (PCP) molecules, as reviewed in [10], [11]. Several mutants of ciliary proteins, in particular proteins for the IntraFlagellar Transport (IFT), show severe defects in forebrain development. The cobblestone mutant, a hypomorphic allele of the IFT gene *Ift88*, has severe defects in the formation of dorsomedial telencephalic structures, and abnormal AP and DV patterning. In this mutant, Gli3 proteolytic processing is reduced and an upregulation of canonical Wnt signalling in the neocortex and in the caudal forebrain can be observed [12]. Inactivation of *Ift172* leads to a global brain-patterning defect through the action of FGF8 signalling at the mid-hindbrain boundary, demonstrating a crucial role in primary cilia formation during development [13]. *Alien* (*aln*) is a mutation in the *Ttc21b* gene, which encodes the complex A protein IFT139 that is important for retrograde IFT. *Aln* mutant mice show loss of the dorsal cortex, DV patterning defects and lack of a clear distinction between the telencephalon and diencephalon mainly due to an upregulation of *Shh* signalling in the diencephalon [14]. A recent study on the role of the ciliopathy gene *Ftm* (*Rpgrip1l*) in brain development demonstrates that olfactory bulb morphogenesis depends on primary cilia [15]. Furthermore, loss of *Kif3a*, a kinesin involved in the IntraFlagellar Transport leads to the degeneration of primary cilia, and disruption of Gli3 processing in the cerebral cortex [16]. Taken together these studies illustrate a critical role for ciliary intraflagellar proteins during forebrain development [17]. However, little is known on the role of ciliary basal body proteins during forebrain development.

Ciliary dysfunction is associated with pathologies named “ciliopathies”. Oral-facial-digital type I syndrome (OFDI; OMIM 311200) is an X-linked dominant developmental ciliopathy with lethality in males. Female patients present malformations of the oral cavity, face, digits and CNS defects with a high degree of phenotypic variability observed in affected females even within the same family, possibly due to X-inactivation [18], [19], [20]. *OFD1*, the gene responsible for this genetic disorder, encodes a protein localized at the basal body of primary cilia [21], [22], [23]. Inactivation of the gene indicates that Ofd1 is required for primary cilia formation at the embryonic node and for left-right axis specification [24]. CNS abnormalities such as agenesis of the corpus callosum, intracerebral cysts/porencephaly, gray matter heterotopias, and cerebellar malformations are present in about 50% of OFDI patients [18]. In more recent years mutation in the *OFD1* transcript have also been identified in patients with Joubert syndrome, a ciliopathy characterized by extensive neuropathological findings [25]. However whether and how OFD1 acts during brain development is still unknown.

To elucidate the role of the ciliary basal body protein Ofd1 in forebrain development, we assessed the neurological phenotype observed in Ofd1 mutant animals. Our data show that Ofd1 controls DV patterning of the forebrain and elongation of ciliary axonemes during development, but not at post-natal stages. In Ofd1 mutant embryos the Shh pathway and apico-basal cell polarity result affected leading to severe patterning and growth defects. Moreover, our study indicates that Ofd1 functions after docking and before elaboration of the axoneme during corticogenesis.

## Results

### Brain phenotypic variability in *Ofd1* heterozygous female embryos

**Figure 1:**
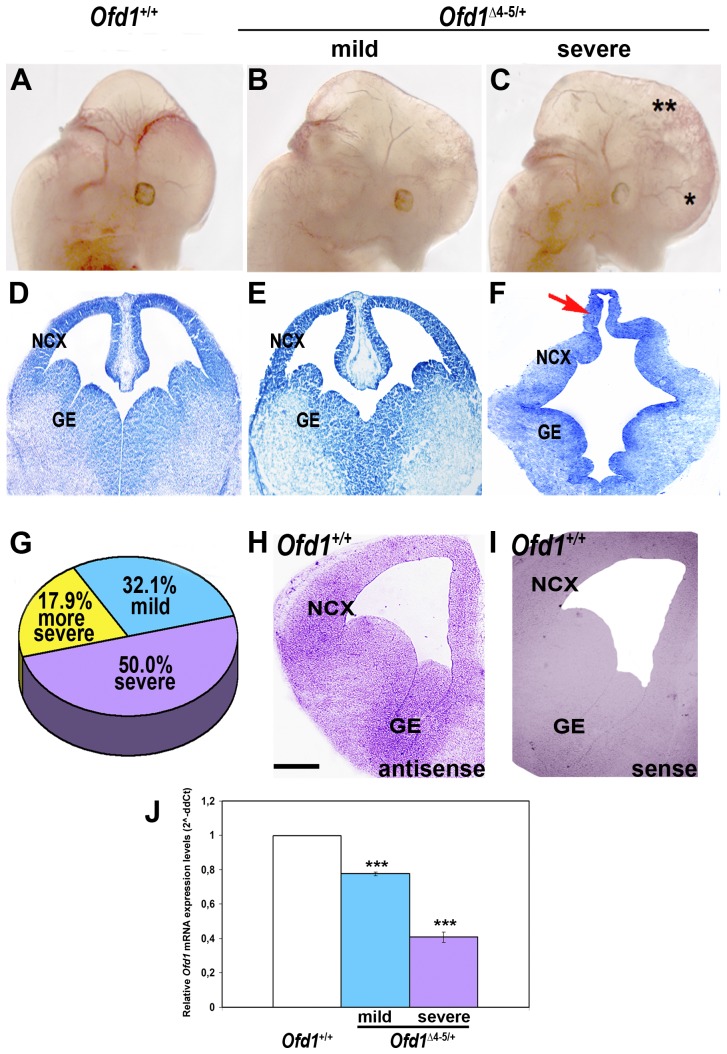
Brain morphology and architecture in wild-type and *Ofd1* mutant females at E12.5. Lateral view of *Ofd1* ^+/+^ wild-type (A) and *Ofd1^Δ^* ^4–5/+^ heterozygous females at E12.5 (B, C). Due to X-inactivation, heterozygous females show a high degree of variability ranging from a mild (B) to a severe (C) neurological phenotype, and an even more severe phenotype (data not shown). In the severe phenotype (C) mutant heads are enlarged, especially at the level of forebrain (*) and midbrain (**). Scale bars: 1 mm. Nissl staining on E12.5 coronal sections of *Ofd1* ^+/+^ wild-type (D) and *Ofd1^Δ^* ^4–5/+^ mutant females (E–F). The mild phenotype (E) shows a structure similar to the wild-type (D). The severe phenotype (F) displays a disorganization of the brain architecture. The presumptive cingulate and hippocampal neuroepithelium fail to normally invaginate and protrude dorsally (red arrow in F). Dorsal is upwards, ventral is downwards. Scale bars: 300 µm. NCX: neocortex. GE: ganglionic eminences. A pie chart indicates the percentage of different neurological phenotypes (G). ISH studies demonstrated that *Ofd1* transcript is expressed in the neocortex (NCX) and in the ganglionic eminences (GE) at E12.5 (H). No signal was detected for the sense riboprobe even after a long incubation time of the sample in staining solution (I). Scale bars: 300 µm. Dorsal is upwards, ventral is downwards. Quantitative RT-PCR is performed upon mRNA extraction from E12.5 total brain (J; ***p<0.01). Error bars indicate standard error of the mean.

To investigate the role of Ofd1 during embryonic development, we have previously generated a mouse model with ubiquitous inactivation of the *Ofd1* transcript [24]. *Ofd1*-knockout animals reproduce the main features of the human disease, albeit with increased severity, possibly due to differences of X-inactivation patterns between human and mouse [19], [26]. In mouse the gene is X-inactivated and female heterozygous mice are mosaics of *Ofd1*-expressing and *Ofd1*-non-expressing cells. In fact, in heterozygous females one wild type or one mutant allele is transcribed in each cell. In *Ofd1*-knockout animals, transcription of the mutant allele leads to the production of an aberrant mRNA encoding a truncated protein of 106 aa. Thus, cells expressing the mutant allele are *Ofd1*-non-expressing cells given that no functional protein is produced [24]. Hemizygous male mutants (*Ofd1* ^Δ4–5/y^) die at E11.5 while heterozygous females (*Ofd1^Δ^* ^4–5/+^) die at birth. To investigate whether Ofd1 is involved during brain patterning, we analyzed heterozygous females (*Ofd1^Δ^* ^4–5/+^) at E12.5, a developmental stage at which the two brain hemispheres are well formed, and the morphological divisions between the dorsal and ventral telencephalon are clearly visible in wild-type animals. Because of the process of X-inactivation we observed a high degree of variability in the phenotype ranging from a mild to a severe phenotype (Figure 1A–C). We performed Nissl staining on E12.5 coronal sections of *Ofd1* ^Δ4–5/+^ female mutants. In mutants with the mild phenotype (Figure 1E) the same morphological structures of wild-type animals could be easily identified (Figure 1D), differently to the severe phenotype where the different brain structures were dramatically affected (Figure 1F). In these embryos, we observed a pronounced disorganization of the dorsal telencephalon in which the medial structures fail to invaginate and tend instead to protrude dorsally (red arrow in Figure 1F). Moreover, mutant embryos showed an abnormally large ventricle and an apparent expansion of the ventral ganglionic eminences (Figure 1F). We performed a macroscopical analysis on 140 heterozygous mutant females at E12.5. Forty-five embryos (32.1%) presented a mild phenotype where the morphology, although abnormal, was better preserved. Seventy embryos (50.0%) showed a severe phenotype that displayed an abnormal brain shape, particularly evident in the forebrain (Figure 1C). Twenty-five embryos (17.9%) showed an even more severe phenotype with a very soft and shapeless brain difficult to handle for experimental manipulation and thus not further characterized (Figure 1G).

Our previous studies revealed that Ofd1 was expressed in the CNS during embryonic development [21] (see also http://www.genepaint.org/Frameset.html for additional data). *In situ* hybridization confirmed that *Ofd1* is expressed both in dorsal and ventral telencephalon with higher levels in the developing cortex and medial ganglionic eminence (MGE) (Figure 1H–I). To correlate the brain phenotype to the expression levels of *Ofd1*, we performed *Ofd1* mRNA expression analysis on total brain of *Ofd1^Δ^* ^4–5/+^ mutant mice by RT-PCR using primers that detect exclusively the wild type *Ofd1* mRNA but not the mutant one. We demonstrated that *Ofd1* mRNA expression levels were reduced by 23% in the mild phenotype, while it was reduced by 60% in the severe phenotype, indicating that the severity of the brain phenotype was due to the percentage of cells carrying the active or inactive mutated X chromosome, and thus to the degree of chimaerism observed in Ofd1 heterozygous females for the X-inactivation phenomenon (Figure 1J).

### Dorsal-ventral patterning is affected in the telencephalon of *Ofd1^Δ^* ^4–5/+^

**Figure 2:**
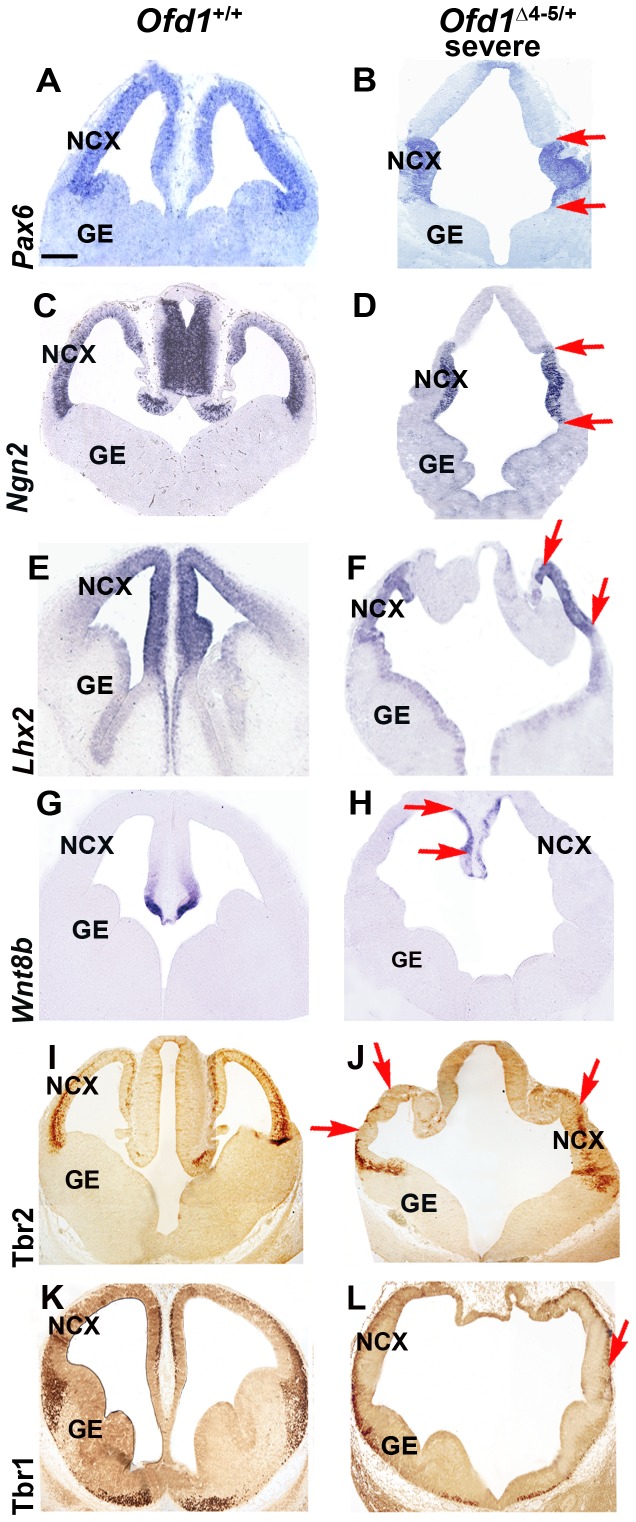
Markers of the dorsal telencephalon are preserved in the absence of Ofd1. *Pax6* and *Ngn2* expression in the forebrain of wild-type (A, D) and *Ofd1* mutants (B, C, E, F) analyzed by ISH on coronal sections at E12.5. These transcripts are normally expressed in *Ofd1* ^+/+^ wild-type and *Ofd1^Δ^* ^4–5/+^ heterozygous females in the dorsal part of telencephalon. In *Ofd1^Δ^* ^4–5/+^ heterozygous females displaying a severe phenotype expression was detected exclusively in the presumptive neocortex (red arrows in B, D), but was absent in the dorsomedial regions. mRNA expression of *Lhx2* was detected in the dorsal telencephalon in a high dorsomedial to low lateral gradient (Figure 2E) [32]. Similarly to *Pax6* and *Ngn2*, *Lhx2* expression lost its expression gradient, but was still maintained in the malformed cortex (red arrows in Figure 2F), and not ectopically expressed in the protruded dorsomedial structure in severely affected O*fd1^Δ^* ^4–5/+^ mutant embryos. mRNA expression of *Wnt8b* was detected in dorsomedial cortical structures, but not in the cortical primordium (Figure 2G) [33]. Severely affected O*fd1^Δ^* ^4–5/+^ mutant embryos showed only a slight expansion of *Wnt8b* expression dorsomedially (red arrows in Figure 2H), indicating that the abnormally protruded dorsomedial structure in mutant embryos has not a cortical origin. Immunohistochemical analysis for Tbr2 reveals that its expression is still maintained in severely affected *Ofd1^Δ^* ^4–5/+^ heterozygous females (I, J) although some areas lack Tbr2 expression (red arrows in J). Similarly, immunohistochemical analysis for Tbr1 reveals that its expression is mainly preserved in *Ofd1^Δ^* ^4–5/+^ heterozygous females with a severe phenotype (K, L) although Tbr1-negative patches can be detected (red arrows in L). Dorsal is upwards, ventral is downwards. Scale bars: 100 µm. NCX: neocortex. GE: ganglionic eminences.

To investigate the molecular basis of the brain defects observed in *Ofd1^Δ^* ^4–5/+^ mutants, we analyzed the expression pattern of several genes involved in different phases of forebrain development. In particular, the dorsal and the ventral telencephalon differ in the expression of distinct markers. The proneural transcription factor *Paired box gene 6 (Pax6)* and *Neurogenin 2* (*Ngn2*) are normally expressed in the dorsal part of the telencephalon where they play a critical role in cortical development [27], [28], [29], [30], [31]. In the wild-type telencephalon, *Pax6* is normally expressed in the cortex with a high lateral to low dorsomedial expression pattern, whereas expression is largely absent from the ventral telencephalon (Figure 2A). In *Ofd1^Δ^* ^4–5/+^ heterozygous females displaying a mild phenotype, *Pax6* was normally expressed in the dorsal telencephalon (data not shown), whereas in *Ofd1^Δ^* ^4–5/+^ mutants with a severe phenotype the expression gradient was lost and *Pax6* became restricted to the morphologically abnormal cortex (red arrows in Figure 2B). No or very low expression was detected in the protruded dorsomedial structure (Figure 2B). Similarly, *Ngn2* expression pattern was normally confined to the cortex in O*fd1^Δ^* ^4–5/+^ mutant embryos displaying both mild and severe phenotypes (data not shown and red arrows in Figure 2D) but absent in dorsomedial telencephalon of severely affected mutant brains. Thus, *Ofd1* mutants displaying different severities of the brain phenotype (mild or severe) showed similar restricted expression pattern of cortical markers, suggesting that the dorsal telencephalon has maintained its cortical regional identity. To further understand the molecular fate of the dorsomedial protruded structures, we analyzed the pattern of *Lhx2*, which is normally expressed in the dorsal telencephalon in a high dorsomedial to low lateral gradient (Figure 2E) [32]. Similarly to *Pax6* and *Ngn2*, *Lhx2* expression has lost its expression gradient, but is still maintained in the malformed cortex, and not ectopically expressed in the protruded dorsomedial structure in severely affected *Ofd1^Δ^* ^4–5/+^ mutant embryos (red arrows in Figure 2F). Finally, we assessed the expression of *Wnt8b*, which is expressed in dorsomedial cortical structures, but not in the cortical primordium (Figure 2G) [33], [34]. Severely affected O*fd1^Δ^* ^4–5/+^ mutant embryos showed only a slight expansion of *Wnt8b* expression dorsomedially (red arrows in Figure 2H), indicating that the abnormally protruded dorsomedial structure in mutant embryos has not a cortical origin.

To investigate whether cortical progenitors are able to resume a normal differentiation program in the absence of Ofd1 function, we performed immunohistochemistry with Tbr2, a marker for intermediate cortical progenitors [35], [36], and Tbr1, which labels the first post-mitotic cortical neurons (Figure 2I–L) [37], [38]. Strikingly, expression of Tbr2 and Tbr1 was maintained in severely affected O*fd1^Δ^* ^4–5/+^ mutant embryos, indicating that corticogenesis is preserved in the presence of a morphological malformed cortical primordium (Figure 2J, L). Nevertheless, Tbr2-negative areas, and to a less extent Tbr1-negative patches, were observed in mutant embryos, suggesting that in some restricted regions cortical progenitors failed to undergo proper differentiation (red arrows in Figure 2J, L).

**Figure 3:**
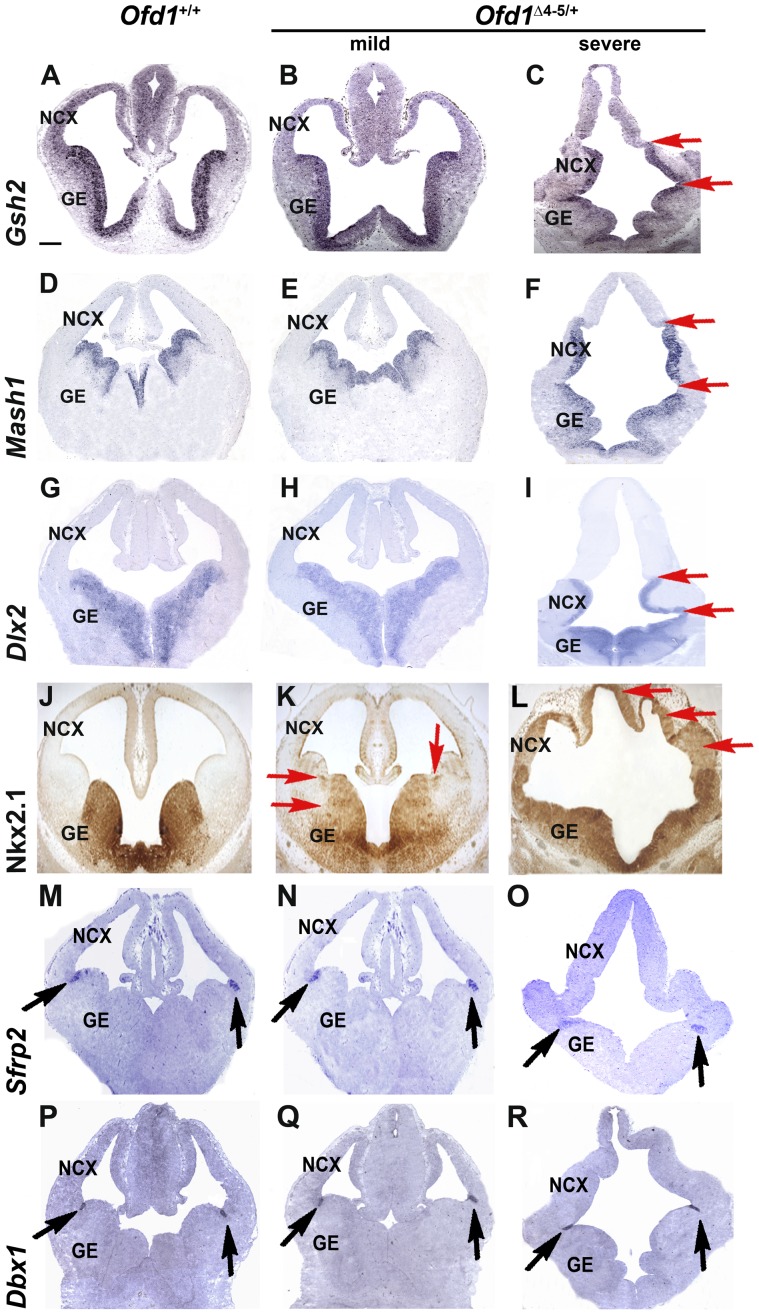
Markers of the ventral telencephalon are ectopically expressed in the dorsal telencephalon of Ofd1 mutant embryos. ISH analysis showed that *Gsh2*, *Mash1* and *Dlx2* genes are all expanded dorsally into the neocortex of *Ofd1^Δ^* ^4–5/+^ heterozygous females with a severe phenotype (red arrows in C, F, I) compared to *Ofd1* ^+/+^ wild-type embryos (A, D, G). Immunohistochemical analysis for the ventral marker Nkx2.1 reveals an ectopic expression in the dorsal part of the brain with a severe phenotype (L) when compared to *Ofd1* ^+/+^ wild-type embryos (J). We do not observe any difference in the expression pattern of ventral markers on brain sections of Ofd1 mutants displaying a mild phenotype (B, E, H) with the exception of Nkx2.1, which is slightly mis-expressed in the LGE (red arrows in K) and it is ectopically induced at lower levels in the dorsal telencephalon (red arrows in L). The analysis of the expression pattern of *Sfrp2* and *Dbx1* genes at the boundary between pallial and subpallial zones (PSBP) demonstrated that these transcripts show a normal expression domain in *Ofd1^Δ^* ^4–5/+^ heterozygous females (black arrows in N, O, Q, R) when compared to wild-type animals (black arrows in M, P). Dorsal is upwards, ventral is downwards. Scale bars: 150 µm. NCX: neocortex. GE: ganglionic eminences.

To assess whether ventral identity was preserved in the absence of Ofd1, we analyzed the expression pattern of markers specific for the ganglionic eminences (GE). The homeodomain gene *Gsh2* and the neural gene *Mash1* are expressed in the progenitor population of the GE and are involved in the maintenance of the molecular identity of this region during development [39], [40], [41], [42], [43]. Strikingly, we found that *Gsh2* and *Mash1* were ectopically expressed in the neocortex of severely affected O*fd1^Δ^* ^4–5/+^ mutant embryos (red arrows in Figure 3C, F), whereas in mildly affected O*fd1^Δ^* ^4–5/+^ mutant animals expression of *Gsh2* and *Mash1* was not altered (Figure 3B, E).

To provide more information on the ventral cell types ectopically located in the dorsal telencephalon, we analyzed the cortical interneuron marker *Dlx2* expressed in the subventricular zone of the lateral (L) GE and medial (M) GE, and Nkx2.1, restricted to the MGE and preoptic area (POA) [44], [45], [46]. High mRNA expression of *Dlx2* was detected in the presumptive neocortical region of severely affected *Ofd1^Δ^* ^4–5/+^ mutant embryos (red arrows in Figure 3I), differently from mildly affected O*fd1^Δ^* ^4–5/+^ mutants (Figure 3H), which show a similar pattern to wild-type embryos (Figure 3G). Immunohistochemical analysis of Nkx2.1 demonstrated that in wild-type *Ofd1* ^+/+^ embryos and in mutant females with a mild phenotype, protein expression was confined to the MGE and POA, although the expression boundary between MGE and LGE was blurred in the mild phenotype and patches of Nkx2.1-positive cells were detected in the LGE (red arrows in Figure 3K), suggesting that the LGE might have partially acquired an MGE-fate. This is exacerbated in the severely affected O*fd1^Δ^* ^4–5/+^ mutant embryos, in which high expression of Nkx2.1 is ectopically induced along the entire ventral telencephalon and at lower levels in the dorsal telencephalon (red arrows in Figure 3L). Ectopic expression of ventral markers in dorsal telencephalon might suggest a lack of morphological division between dorsal and ventral regions of the telencephalon. However, no differences in the expression of pallial-subpallial boundary (PSPB) markers such as *Sfrp2* and *Dbx1* [47], [48] were detected in both mildly and severely affected *Ofd1^Δ^* ^4–5/+^ mutant embryos (black arrows in Figure 3M–R), suggesting that ectopic activation of ventral markers in dorsal telencephalon is independent of the presence of a PSPB boundary.

Taken together these data suggest that Ofd1 plays an important role in DV patterning of the telencephalon and particularly in restricting ventral telencephalic fate during forebrain neurogenesis.

### Shh signalling and Gli3 protein processing are defective in the *Ofd1^Δ^* ^4–5/+^ forebrain

In the absence of Sonic Hedgehog (Shh), the transmembrane protein Patched 1 (Ptch1) inhibits Smoothened (Smo) in transducing the signal, and as a result, the full-length activator form of Gli3 (Gli3^FL^), a transcriptional effector of the *Shh* signaling pathway, is proteolytically cleaved into the repressor form Gli3^R^. The binding of Shh to Ptch1 induces the release of Smo, which in turn inhibits Gli3 processing. As a result of Shh pathway activation, the Gli3 activator induces downstream targets. Some of the abnormalities observed in *Ofd1* mutant animals resemble defects of the extra-toes *Gli3^Xt−J/Xt−J^* mutant in which Gli3 is not expressed due to a deletion within the 3′ end of the gene [49], [50], [51], [52], [53].

**Figure 4:**
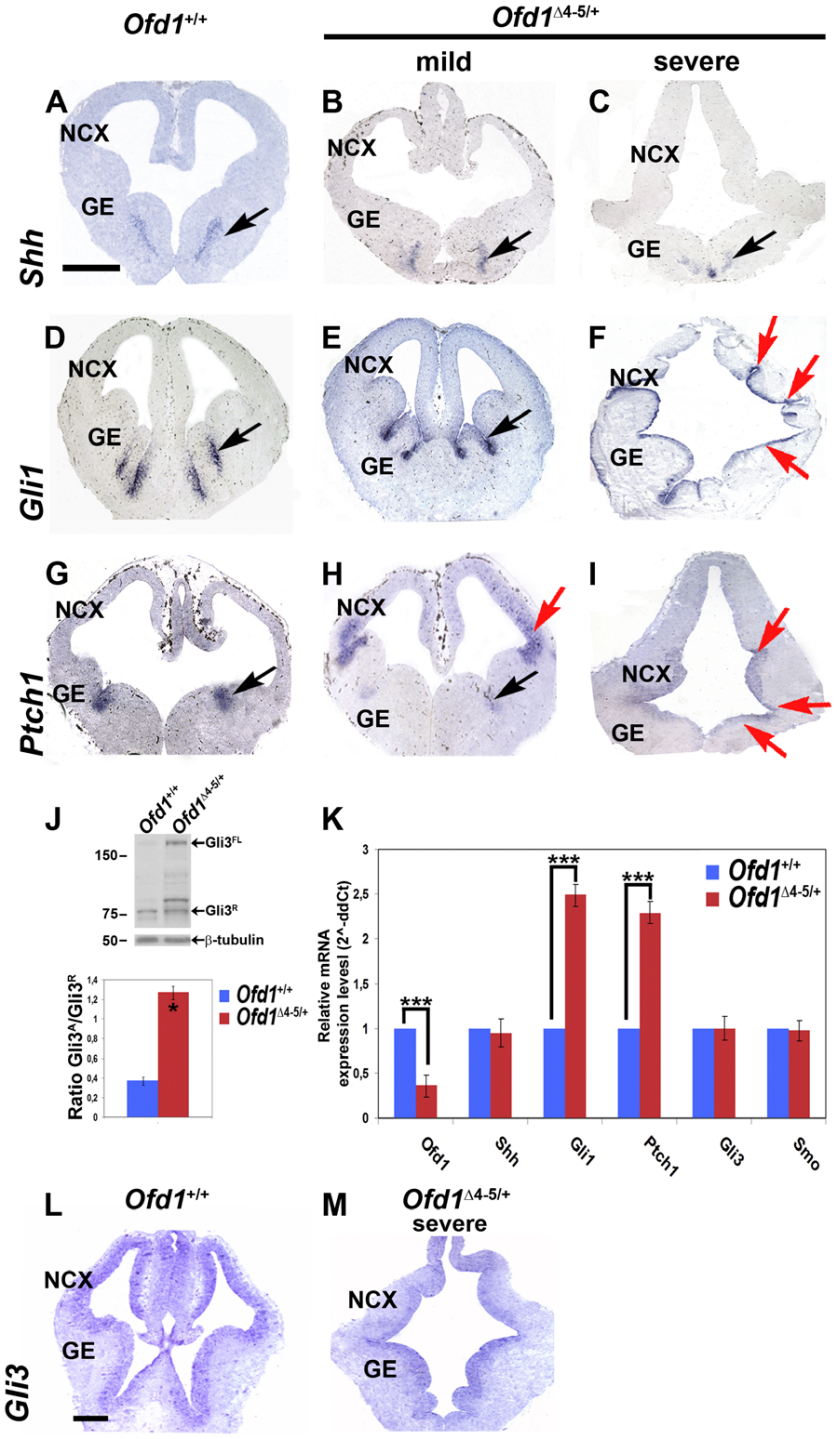
Shh signalling is altered in the developing forebrain of *Ofd1^Δ^* ^**4–5/+**^ **embryos.** ISH showed a normal expression pattern of *Shh* in *Ofd1^Δ^* ^4–5/+^ heterozygous females (black arrows in B, C) when compared to *Ofd1* ^+/+^ wild-type embryos (black arrow in A). *Gli1* displays an ectopic expression in the neocortex in mutants with a severe phenotype (red arrows in F) while it is confined to the ventral part in *Ofd1* ^+/+^ wild-type embryos (black arrow in D) and *Ofd1^Δ^* ^4–5/+^ heterozygous females with a mild phenotype (black arrow in E). *Ptch1* expression is upregulated in the neocortex in both mutants with a mild (red arrow in H) and severe phenotype (red arrows in I). Dorsal is upwards, ventral is downwards. Scale bars: 200 µm. NCX: neocortex. GE: ganglionic eminences. Western blot analysis of Gli3 protein on E12.5 *Ofd1* ^+/+^ wild-type and *Ofd1^Δ^* ^4–5/+^ heterozygous brains with a severe phenotype. An increase level of the larger isoform 190 kDa Gli3^FL^ isoform is observed in Ofd1 mutant animals when compared to controls, thus indicating an impairment during Gli3 processing (J). Quantification of the ratio of Gli3^FL^ versus Gli3^R^ indicates a 3.4-fold increase in the mutant animals (J). Asterisk (*) denotes statistically significant changes with *p*<0.05. Quantitative RT-PCR is performed upon mRNA extraction from E12.5 total brain (K; ***p<0.01). Error bars indicate standard error of the mean. ISH analysis showed that no difference in Gli3 mRNA expression pattern was observed in *Ofd1^Δ^* ^4–5/+^ heterozygous females with a severe phenotype (M) when compared to *Ofd1* ^+/+^ wild-type embryos (L). Dorsal is upwards, ventral is downwards. Scale bars: 100 µm. NCX: neocortex. GE: ganglionic eminences.

We thus investigated whether Shh signalling was perturbed in the forebrain of *Ofd1* mutant embryos by analyzing the expression pattern of *Shh*, *Ptch1* and *Gli1*, which encode for the ligand, the receptor and the downstream target of Shh signalling, respectively. In both severely and mildly affected E12.5 mutants, Shh expression remained confined to the pre-optic region with no evidence of ectopic dorsal spread (black arrows in Figure 4A–C). In contrast, the mRNAs of both *Gli1* and *Ptch1* were ectopically expressed in the dorsal telencephalon in mutants with the severe phenotype (red arrows in Figure 4F, I). However, while *Gli1* expression was not altered in the mild phenotype (black arrow in Figure 4E), restricted expression of *Ptch1* in the intraganglionic sulcus was now shifted to the PSPB boundary and lateral cortex of mildly affected *Ofd1* mutant embryos (red arrow in Figure 4H). Thus, in the absence of Ofd1, Shh-induced targets were ectopically expressed in the dorsal telencephalon leading to abnormal Shh signaling in the developing cortex.

Since a misregulation of Shh pathway could be due to altered proteolytic processing of Gli3 as reported in numerous ciliary mutants [15], [54], [55], [56], [57], [58], [59], [60], we assessed Gli3 processing in *Ofd1* mutants. We performed western blot analysis on protein lysates from the forebrain of wild-type and mutant embryos, by using an antibody that recognizes both the full-length and repressor forms of Gli3. This analysis revealed that the ratio between Gli3^FL^ and Gli3^R^ levels was increased in the forebrain of mutant animals with a 3.4-fold increase of the Gli3^FL^ form versus the Gli3^R^ form in *Ofd1^Δ^* ^4–5/+^ heterozygous female showing a severe phenotype, as demonstrated by densitometric analysis (Figure 4J).

To further confirm abnormal Shh signalling in *Ofd1^Δ^* ^4–5/+^ mutant females, we performed quantitative mRNA expression analysis on total brains of E12.5 embryos by RT-PCR (Figure 4K). First, we analyzed mRNA expression levels of *Ofd1* in total brains with a severe phenotype and confirmed a 60% decrease of *Ofd1* transcript. While mRNA expression levels of *Shh* were not modified, *Gli1* and *Ptch1* expression levels were upregulated (Figure 4K), in accordance with the above-mentioned molecular markers analysis. Interestingly, no changes in expression levels for *Smo* were detected in mutant embryos displaying a severe phenotype (Figure 4K), indicating that Ofd1 acts mainly on the expression of the Ptch1 receptor, but not on Smo, and that alteration of Gli3 function was due to altered protein processing rather than to a control at the transcriptional level. Since mRNA levels of Gli3 were not altered in Ofd1 mutant embryos, as also confirmed by in situ hybridization experiment (Figure 4L–M), we hypothesize that the full-length isoform of Gli3 is more stable than the processed one ultimately leading to a total increase of the amount of Gli3. These data support previous findings reported in another study on the role of primary cilia during corticogenesis [12]. Hence, *Ofd1* mutant females with a severe phenotype show an upregulation of the Shh pathway in the dorsal telencephalon most likely due to defective Gli3 processing.

### Ofd1 is essential for ciliogenesis in the embryonic brain but is dispensable in the post-natal cortex

**Figure 5:**
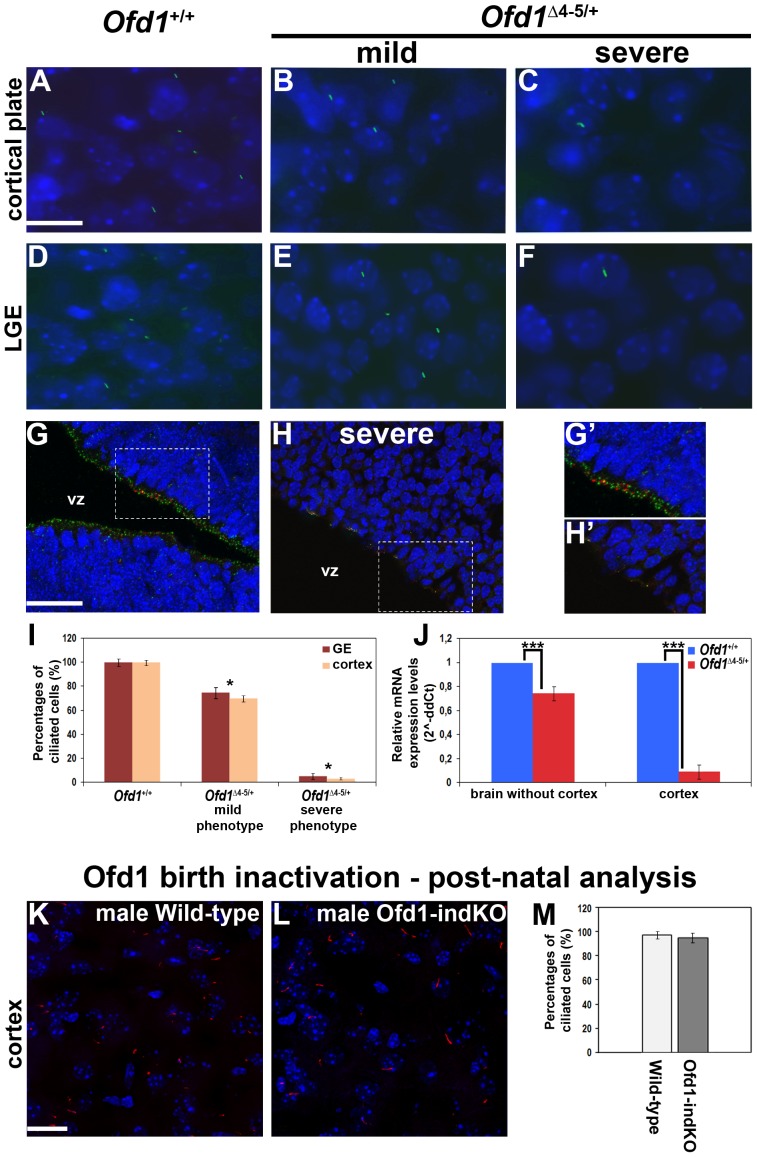
The number of neuronal cilia is reduced in Ofd1 mutant embryos at E12.5 while cilia are not affected upon Ofd1 inactivation at E18.5. A–J. Immunofluorescence analysis by anti-Adenylyl cyclase type III (green) in *Ofd1* ^+/+^ wild-type embryos (A, D), *Ofd1^Δ^* ^4–5/+^ heterozygous females with a mild phenotype (B, E) and in *Ofd1^Δ^* ^4–5/+^ heterozygous females with a severe phenotype (C, F) at E12.5. Nuclei are counterstained with DAPI (blue). In both the cortical plate (A, B, C) and the lateral ganglionic eminences (LGE) (D, E, F) the number of cilia is dramatically reduced in embryos with a severe phenotype (C, F). Scale bars: 10 µm. Immunofluorescence analysis by anti-Adenylyl cyclase type III (green labels the cilium) and anti-γ tubulin (red labels the basal body) specific for centrosomes in *Ofd1* ^+/+^ wild-type embryos (G), and in *Ofd1^Δ^* ^4–5/+^ heterozygous females with a severe phenotype (H) at E12.5. Dashed rectangles designate enlarged areas indicated in panels G′ and H′ showing primary cilia at the ventricular surface of the dorsal telencephalon (G′, H′). Histograms indicate the percentage of ciliated cells in the cortex and in the GE (I). In both cases the number is significantly reduced in the severe phenotype at E12.5. Asterisk (*) denotes statistically significant changes with *p*<0.05. Quantitative RT-PCR is performed upon mRNA extraction from E12.5 brain. The analysis is performed on isolated cortex and on the remaining part of the brain (J; ***p<0.01, Student's test). Error bars indicate standard error of the mean. K–M. Immunofluorescence analysis by anti-Adenylyl cyclase type III (red) in male wild-type mice (K) and male Ofd1-indKO mice (L) at P30. Nuclei are counterstained with DAPI (blue). Neuronal primary cilia are still present in mutant animals analyzed at P30 in which Ofd1 inactivation was induced at E18.5 and. Scale bars: 20 µm. Histograms indicate no difference in the percentages of ciliated cells in male wild-type mice and male Ofd1-indKO mice (*M*).

To evaluate the effect of *Ofd1* inactivation on cilia formation, we performed immunofluorescence analysis on forebrain sections using the Adenylyl-Cyclase III antibody, which stains specifically the ciliary axoneme of neuronal cells [61], [62]. Strikingly, the number of primary cilia was dramatically reduced in the forebrain and at the apical side of E12.5 telencephalic neuroepithelial cells of severely affected *Ofd1^Δ^* ^4–5/+^ mutants (Figure 5A–H′H). In the ganglionic eminences, 75% and 5% of total cells analyzed were still ciliated in embryos with mild and severe phenotype, respectively (Figure 5I). Differently, in the cortex of mildly affected mutant embryos, 70% of the total cells analyzed were still ciliated, whereas the number was drastically reduced to 3% in those displaying a severe phenotype (Figure 5I). Since we observed a reduction in the number of ciliated cells, we decided to investigate the presence of centrosomes at the bases of ciliary protrusions. We counted the number of centrosomes observed in the immunofluorescence analysis (Figure 5G, H), on different sections (at least three sections per three mice per genotype) and we found no differences between wild-type and severely affected heterozygous females (data not shown). To further understand the nearly total absence of primary cilia in the cortex of severely affected mutants despite the apparent expression levels of *Ofd1* measured in the total brain (residual expression level of 40%, Figure 1J), we dissected the whole brain of wt and *Ofd1^Δ^* ^4–5/+^ severe mutant embryos and performed *Ofd1* mRNA expression analysis by RT-PCR separately on the cortex and on the whole remaining brain (Figure 5J). Strikingly, we found that *Ofd1* mRNA expression levels were drastically reduced (residual expression level of 10%) in the cortex of severe mutants, indicating that the cortex is almost homogeneously deficient for Ofd1. To determine more accurately the extent of Ofd1 inactivation, we performed quantitative RT-PCR with primers that specifically amplify the wild-type *Ofd1* allele on genomic DNA obtained from the cortex and from the whole remaining brain of embryos. The analysis revealed that in *Ofd1^Δ^* ^4–5/+^ severe mutant embryos only 12% (±0.02) of wild-type allele is still present in the cortices while the whole remaining brain yet expresses 86% (±0.03) of wild-type allele. Thus, we found a clear correlation between *Ofd1* expression levels and distribution (cortex versus whole remaining brain) and severity of the phenotype observed in the cortex.

To investigate whether Ofd1 has a developmental stage-dependent role in the forebrain, we analyzed a conditional null mouse model containing the *Ofd1* floxed allele and the tamoxifen-inducible *Cre-recombinase* expressed from the actin promoter (CAGG-creER™) [63]. Efficient deletion of *Ofd1* was induced just before birth by injecting pregnant mothers at E18.5. Our previous studies revealed that expression of the Ofd1 transcript is maintained in the cortex at post-natal stages [21]. Thus, we measured mRNA expression levels through RT-PCR to validate *Ofd1* inactivation at birth and in adult stages. Our results indicate that tamoxifen injection strongly downregulates Ofd1 at P0 (88%±1.5 reduction in mutated cortex compared to the wild-type cortex) and that inactivation is still maintained at P30 (85%±2.2 reduction compared to the wild-type cortex). We next analyzed the presence of primary cilia by using the Adenylyl-Cyclase III antibody on sections from *Ofd1* ^flox/y^ (wild-type) and *Ofd1* ^flox/y^; CAGG-creER™ (hereafter called Ofd1-indKO) animals (Figure 5K, L). Interestingly, *Ofd1-indKO* male mutant mice were still viable 30 days after tamoxifen injection, which allowed us to focus on a post-natal Ofd1 null mouse model. Unexpectedly, similar numbers of primary cilia were detected on cortical neurons of wild-type and *Ofd1-indKO* mice (Figure 5M), suggesting that Ofd1 is dispensable for ciliogenesis in the post-natal cortex. Taken together, these data demonstrate that Ofd1 plays a crucial role in ciliogenesis primarily during embryonic developmental stages.

### Ofd1 is not required for basal body docking and orientation but is crucial for axoneme elongation *in vivo*

**Figure 6:**
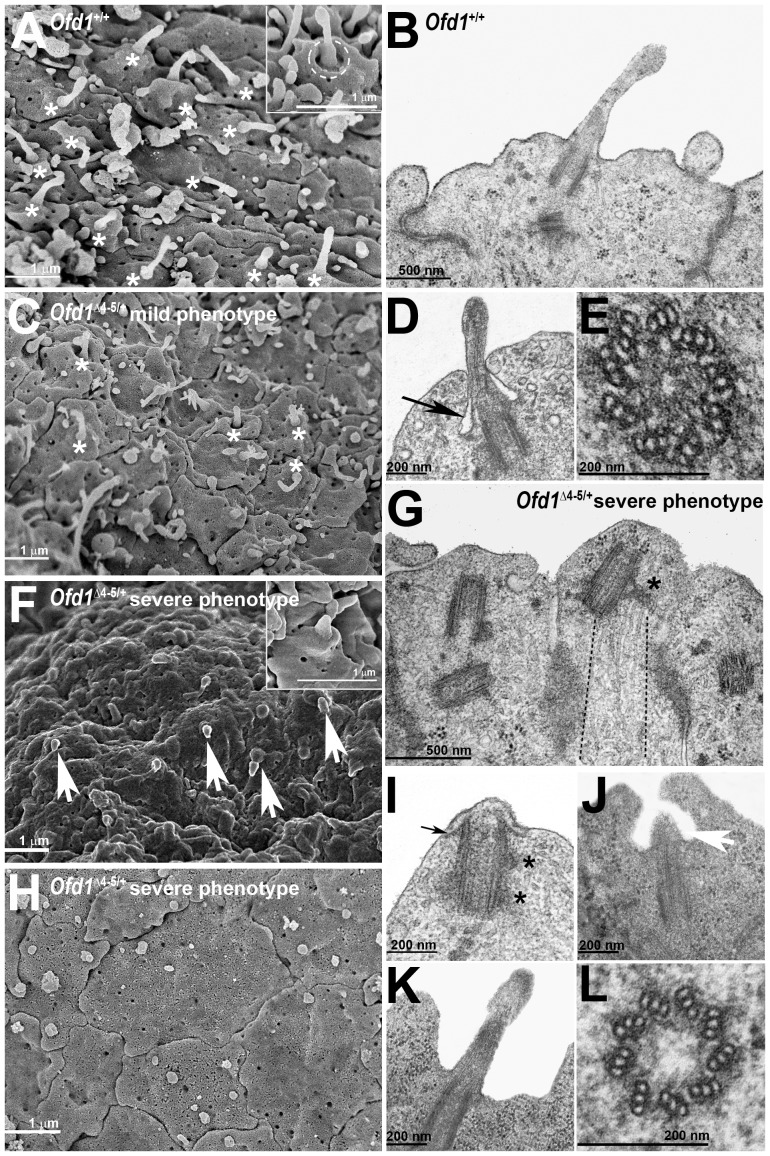
Neuronal primary cilia are severely affected in *Ofd1^Δ^* ^**4–5/+**^ **forebrains.** SEM analysis of neocortex from embryos at E12.5 (A, C, F, H). Numerous primary cilia (white asterisks) are present in *Ofd1* ^+/+^ wild-type neocortex (A). A higher magnification of a primary cilium where the pit is more evident (dashed white circle) is reported in inset *A*. The distribution of primary cilia (white asterisks) is reduced in *Ofd1^Δ^* ^4–5/+^ heterozygous females displaying a mild phenotype (C). In severely affected *Ofd1^Δ^* ^4–5/+^ heterozygous females, we observe regions with short protrusions of membrane indicated by white arrows (F) and regions completely devoid of cilia (H). TEM analysis of neocortex from embryos at E12.5 (B, D, E, G, I, J, K, L). Primary cilium in *Ofd1* ^+/+^ wild-type neocortex (B). Primary cilium in *Ofd1* ^+/+^ wild-type neocortex where the ciliary pocket is evident, as indicated by the black arrow (D). Transverse section of a normal basal body, where the nine triplets of microtubules are easily recognized (E). No ciliary axonemes can be detected in severely affected *Ofd1^Δ^* ^4–5/+^ heterozygous females (G). The basal body in *Ofd1^Δ^* ^4–5/+^ heterozygous females appear mature as indicated by the presence of appendages (black asterisk) on which microtubules are correctly anchored (dashed black lines demarcate the area where microtubules can be observed) (G). The basal body in severely affected *Ofd1^Δ^* ^4–5/+^ heterozygous female appear to be correctly docked as indicated by the presence of the sheath (black arrow) and, as described above, they appear to be mature, given the presence of appendages (black asterisks) (I). Similar to SEM analyses, TEM analyses reveal that severely affected *Ofd1^Δ^* ^4–5/+^ heterozygous female show short protrusions of membrane indicated by white arrow (J). One of the few ciliary axonemes present in a severely affected *Ofd1^Δ^* ^4–5/+^ heterozygous female is showed (K). Transverse section of the basal body in a severely affected *Ofd1^Δ^* ^4–5/+^ heterozygous female showing a normal ultrastructure with nine triplets of microtubules (L).

Given the lack of ciliary axonemes in *Ofd1^Δ^* ^4–5/+^ severely affected mutant embryos, we decided to investigate the ultrastructure of basal bodies to elucidate the role of Ofd1 in ciliogenesis. We performed both Scanning Electronic Microscopy (SEM) and Transmission Electron Microscopy (TEM) analyses on neocortical cells of 3 *Ofd1^+^* ^/+^ wild-type and 3 *Ofd1^Δ^* ^4–5/+^ severely affected mutant embryos. SEM analysis showed numerous primary cilia emerging from a pit in the apical cell surface of *Ofd1^+^* ^/+^ wild-type embryos (Figure 6A). The number of primary cilia was reduced in *Ofd1^Δ^* ^4–5/+^ mutant embryos with a mild phenotype (Figure 6C), while no primary cilia were observed in the cortex of severely affected mutant embryos (Figure 6H), even if a few stunted protrusions were occasionally observed in some cases (Figure 6F). TEM analysis revealed the presence of mature basal bodies and primary cilia (n = 58) with a normal ultrastructure (9×3) in *Ofd1^+^* ^/+^ wild-type animals (Figure 6B, D, E). On the contrary, we observed almost complete lack of ciliary axonemes in *Ofd1^Δ^* ^4–5/+^ mutant females as illustrated in Figure 6G, I, J. At the distal tip of the basal bodies, a short expansion of the membrane could be observed, but this structure was either embedded in the neuroepithelium or much shorter than a primary cilium and, importantly, lacked an axoneme (Figure 6G, I, J). We counted n = 52 mature basal bodies with no protruding primary cilium and only very few basal bodies with primary cilia (n = 3) in *Ofd1^Δ^* ^4–5/+^ severe mutant embryos (Figure 6K). The basal body appeared correctly orientated with a normal ultrastructure (Figure 6L). The appendages are an important site of microtubule anchoring with characteristic TEM appearances depending on the plane of section [64]. In mutant embryos, distal appendages were evident by TEM (Figure 6G, I). Moreover, loss of Ofd1 did not affect microtubule anchoring (Figure 6G). In addition, we did not observe any difference in length of basal bodies in *Ofd1^Δ^* ^4–5/+^ mutant embryos. In fact, in the cortex of wild-type embryos the average length for basal bodies (n = 35) was 360±50 nm and in *Ofd1^Δ^* ^4–5/+^ severe mutant embryos it was 369±73 nm for n = 26 basal bodies analyzed.

Our data indicate that Ofd1 is mainly involved in ciliary axoneme elongation in the developing forebrain but not in basal body orientation, docking and maturation.

### Cytoskeletal organization and apico-basal cell polarity are affected in *Ofd1^Δ^* ^4–5/+^

**Figure 7:**
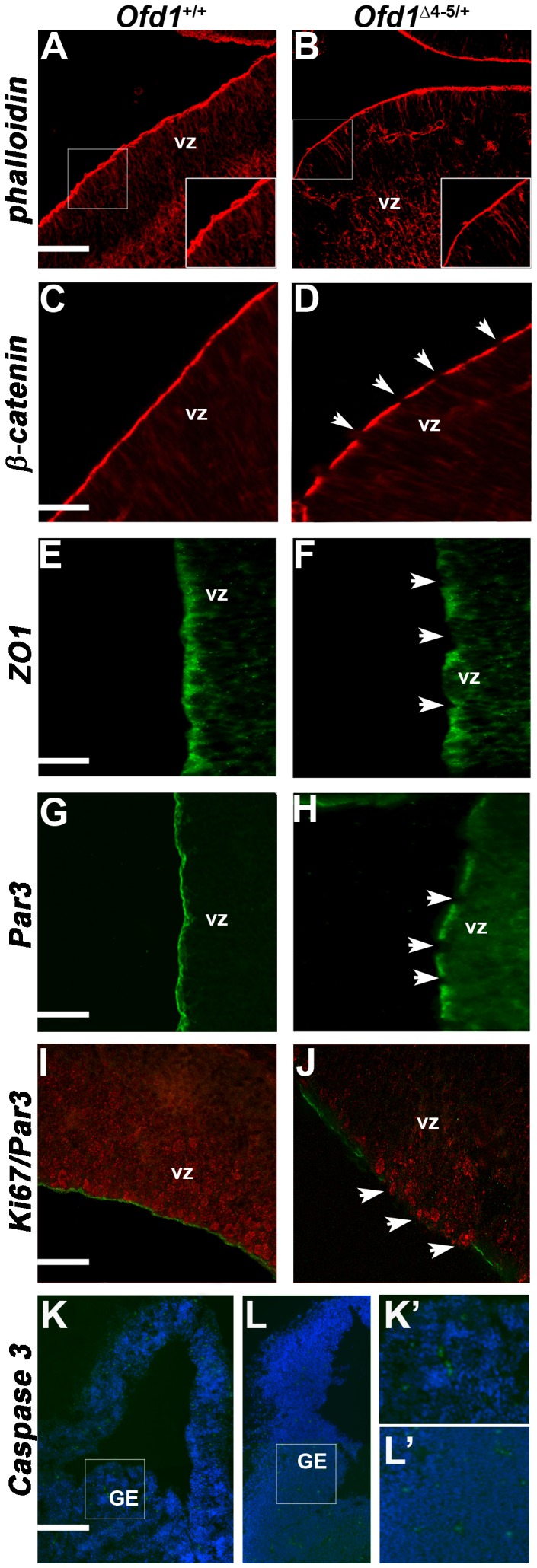
Cytoskeletal organization and cell polarity are altered in *Ofd1^Δ^* ^**4–5/+**^ **embryos.** Staining for F-actin with fluorescent phalloidin reveals a reduced signal and actin disorganization at the cell apex in severely affected *Ofd1^Δ^* ^4–5/+^ mutants (B) with respect to wild-type *Ofd1^+^* ^/+^ embryos (A). Scale bars: 200 µm. A higher magnification of the actin boundary indicates a reduced thickness in mutant embryos (inset B) compared to wild-type *Ofd1^+^* ^/+^ embryos (inset A). Immunofluorescence with β-catenin reveals a continuous band in wild-type embryos (C) but a punctuate and discontinuous band in *Ofd1^Δ^* ^4–5/+^ severe mutants (D). Scale bars: 100 µm. Immunofluorescence analysis of ZO1, a marker of tight junctions, shows a discontinuous pattern along the margin of the ventricular surface (F) compared to wild-type embryos (E). Scale bars: 50 µm. Immunostaining of Par3 with a discontinuous pattern along the cells lining the ventricular zone in *Ofd1^Δ^* ^4–5/+^ severe mutant embryos (H) compared to wild-type embryos (G). Scale bars: 50 µm. White arrows in panels D, F, H indicate expression discontinuity along the apical membrane. Immunostaining of Ki67 (in red) and Par3 (in green) indicate reduced Ki67 expression and presence of abnormally located cortical progenitors, which tend to protrude outside the ventricular zone in *Ofd1^Δ^* ^4–5/+^ severe mutant embryos (J) compared to wild-type embryos (I). Scale bars: 200 µm. vz: ventricular zone. Immunofluorescence analysis of Caspase 3 revealed no difference in the number of apoptotic cells in wild-type embryos (K) and in *Ofd1^Δ^* ^4–5/+^ severe mutant embryos (L). Dashed rectangles designate enlarged areas indicated in panels K′ and L′ showing apoptotic cells in the ganglionic eminence (GE) region. Scale bars: 500 µm.

Previous studies reported the importance of apical actin enrichment during ciliogenesis [65], [66]. Thus, we first analyzed the distribution of F-actin by staining with fluorescent phalloidin. In *Ofd1^Δ^* ^4–5/+^ severe embryos we observed a reduction of actin staining at the cell apex (Figure 7A–B), consistent with defective ciliogenesis (Figure 5I). To understand further cytoskeletal rearrangements, we analyzed the distribution of β-catenin, a cell adhesion molecule that anchors the actin cytoskeleton. Immunostaining with β-catenin revealed a continuous band in wild-type embryos (Figure 7C), but a punctuate and discontinuous band in *Ofd1^Δ^* ^4–5/+^ severely affected embryos (arrowheads in Figure 7D), suggesting defective cell adhesion properties in mutant embryos. Hence, we analyzed the expression pattern of ZO1, a marker of tight junctions and consistently, we found a discontinuous pattern along the margin of the ventricular zone in *Ofd1^Δ^* ^4–5/+^ severely affected embryos (arrowheads in Figure 7F) compared to wild-type embryos (Figure 7E). Thus, in *Ofd1* severely affected heterozygous females the apical membrane is discontinuous and shows several gaps along the margin of the ventricular zone suggesting apico-basal cell polarity defects. Several studies demonstrated that cell polarity is regulated by both the Par3/Par6/aPKC complex and the PCP pathway [67], [68]. The polarity protein Par3, crucial for growth and elongation of the primary cilium in epithelial cells [69], is expressed in a discontinuous pattern along the cells lining the ventricular zone of *Ofd1^Δ^* ^4–5/+^ severely affected embryos (Figure 7G, H). Given that Par-complex proteins promote proliferative progenitor divisions in the developing mouse cerebral cortex [70], we performed double immunofluorescence for Par3 and Ki67, a marker for proliferative cells. We observed abnormal expression of Ki67 and most importantly disorganized and disoriented progenitors in the regions devoid of Par3 in *Ofd1^Δ^* ^4–5/+^ severely affected embryos (arrowheads in Figure 7J) with respect to wild-type *Ofd1^+^* ^/+^ embryos (Figure 7I). Thus, abnormal cell polarity at the apical membrane observed in patches along the ventricular zone might correlate with disrupted Tbr2 and Tbr1 expression, as shown in Figure 2H and J, suggesting that abnormal cell polarity and cytoskeletal rearrangements due to defective ciliogenesis might interfere with proper corticogenesis. Finally, we assessed whether cell death was increased in mutant embryos by testing the presence of Caspase 3-positive cells. We found no obvious difference between wild-type and O*fd1^Δ^* ^4–5/+^ mutant embryos in the distribution of dying cells (Figure 7K–L).

## Discussion

### *Ofd1^Δ4–5/+^* mutants display a phenotypic variability

We assessed Ofd1 function during forebrain development and we found that *Ofd1^Δ^* ^4–5/+^ heterozygous female mutants displayed a phenotypic variability that ranges from mild to severe. This is most probably due to the mosaicism expected for the X-inactivation phenomenon in mice, in which the amount of Ofd1 expressed in heterozygous females depends on which allele, and also in which cell-type Ofd1 function is abolished. Once X-inactivation has occurred, all cells derived from a common progenitor maintain the same pattern of inactivation. On the basis of this assumption, we focused our analysis mainly on *Ofd1^Δ^* ^4–5/+^ embryos with a severe phenotype in which Ofd1 inactivation in the cortex is almost complete, as demonstrated by our quantitative analysis. Nevertheless, our study shows that the cell-type-dependent mosaic inactivation of Ofd1, even in the severe phenotype, could lead to variable and mixed cellular phenotypes. For example, we demonstrated that Ofd1 inactivation during mouse embryonic development results in ventralization of the telencephalon, a phenotype also described in other ciliary-specific mutant mice [8]. We found that dorso-ventral patterning of the telencephalon in *Ofd1^Δ^* ^4–5/+^ mutant embryos was severely compromised, as shown by the expansion of ventral markers in the dorsal part of the telencephalon. However, proper cortical markers were also maintained in the dorsal telencephalon of *Ofd1^Δ^* ^4–5/+^ embryos, leading most probably to a presumptive cortex with mixed DV identity. Although we do not know yet the reason of this mixed phenotype, we presume that it might be part of the mosaic cellular inactivation, as described above. Moreover, analysis of primary cilia revealed the nearly complete absence of protruding ciliary axonemes in the cortex of severely affected *Ofd1^Δ^* ^4–5/+^ mutant embryos. In the mild phenotype, we found a “patchy” distribution with some regions devoid of primary cilia and some with protruding structures, in accordance with a less severe phenotype associated, again, to the mosaicism observed for the X-inactivation in female embryos.

### Inactivation of the basal-body protein Ofd1 leads to perturbation of the Shh pathway

It is well established that the Hh pathway is a major regulator of growth and patterning in both invertebrates and vertebrates [71], [72]. Interestingly, the primary cilium and IFT machinery have shown to play an essential and vertebrate-specific role in Hh signal transduction [54], [55], [73], [74], [75], [76], [77]. In fact, several mutants of ciliary proteins, primarily belonging to the IFT machinery, present severe defects in forebrain development coupled to an alteration in Hh signalling. For example, the cobblestone mutant, a hypomorphic allele of *Ift88*, shows severe defects in brain patterning associated to an altered Gli3^FL^ and Gli3^R^ ratio and increased *Gli1* and *Ptch1* expression levels in the ventral telencephalon [12]. Similarly, mutations in *Ift139* lead to DV patterning defects and activation of the *Shh* pathway [14]. Furthermore, *Ift172* mutant embryos show severe brain patterning defects, which are associated to downregulation of the *Shh* pathway and *Gli1* expression [13]. Similarly, in the absence of *Rfx4*, an upstream regulator of *Ift172*, mouse mutants have distinct DV patterning defects in the ventral spinal cord and telencephalon due to aberrant Shh signalling and Gli3 activity [78]. Finally, mouse mutants lacking the ciliopathy gene *Ftm*/*Rpgrip1l* have brain defects due to the reduction of the Gli3^R^ form [15].

However, analysis of the mutant *hennin* phenotype did not correspond to either a simple decrease or increase in the activity of the Hh pathway. Cells requiring the highest Hh activity failed to be specified, whereas cells requiring intermediate Hh activity are found in an expanded domain, a phenotype not described in other mouse mutants [79]. These data show that misregulation of the Shh pathway due to mutations in ciliary proteins does not always follow the same trend (in some cases the pathway is downregulated, in others upregulated), and suggests that each ciliary protein might play different roles in the regulation of the Shh pathway and/or Gli3 processing.

Little is known on the role of basal body proteins in Shh signalling during forebrain development. Our studies show that the basal body protein Ofd1 seems to regulate the Shh pathway differently depending on the tissue and on the developmental stage. In fact, our previous reports demonstrated alterations of the Shh pathway and reduced *Gli1* and *Ptch1* expression levels in limbs deficient for *Ofd1* at E11.5 [80], in the myeloma cell line MM1S inactivated for *Ofd1* [81] as well as in the ventral neural tube of male mutant *Ofd1* embryos at E9.5 [24]. On the contrary, in the brain of *Ofd1* mutants analyzed in the present study we report an activation of the Shh pathway, as demonstrated by increased and ectopic expression of *Gli1* and *Ptch1* in the dorsal telencephalon. We measured no differences in *Shh* and *Smo* expression levels, suggesting that Ofd1 acts downstream of the ligand in the Hh pathway. Consistent with an increase of both *Gli1* and *Ptch1* mRNA expression levels, we showed an increase of the Gli3^FL^ form. Although a recent paper elegantly demonstrated that the Gli3^R^ form is the major player during telencephalic DV patterning in *Ftm* mutants, we cannot exclude that altered Gli3 processing might have a different outcome in *Ofd1* mutants.

### Ofd1 plays a stage dependent role in late phases of ciliogenesis

Ciliogenesis is characterized by several steps. First, the centrosome migrates to the apical cell surface during early cell polarization. Once the centrosome has migrated to and docked with the apical cell surface, it matures to form the basal body. The last phase of ciliogenesis is the axoneme elongation [82]. Our study demonstrates for the first time that Ofd1 participates in axoneme formation during late phases of ciliogenesis after basal body docking and before axoneme elongation. Indeed, ultrastructural analysis of the neocortex of severely affected *Ofd1^Δ^* ^4–5/+^ mutants showed that the basal bodies were mature, correctly docked and orientated, however primary cilia, e.g. axonemes, could not be detected. This is also in accordance with our previous studies. Analysis of primary cilia in the developing limb, in which *Ofd1* complete inactivation becomes effective only by E11.5 in the limb, revealed that although primary cilia do appear, they have a shortened and malformed axoneme [80]. Moreover, no primary cilia could be detected in *Ofd1^Δ^* ^4–5/y^ hemizygous male mutants at the embryonic node, although some were identified in *Ofd1^Δ^* ^4–5/+^ heterozygous female mutants [24]. All these studies strongly suggest a stage-dependent role for Ofd1 in axoneme elongation during ciliogenesis. The different models (*in vitro versus in vivo*) and the stage-dependent role for Ofd1 in axoneme elongation could explain the apparent discrepancy with previous data obtained in murine embryonic stem cells that demonstrate that Ofd1 is a component of the distal centriole and is crucial for the formation of the distal appendages, which is a prerequisite for cilium formation [83], [84].

In our *in vivo* animal model, and in particular during corticogenesis, EM analyses revealed that the basal body length was not affected and that distal appendages correctly formed in the absence of Ofd1. Thus, the basal body protein Ofd1 is not required for basal body migration, docking and maturation but controls axoneme elongation thereby indicating, for the first time, that Ofd1 functions after docking and before elaboration of the axoneme.

To date, the role during post-natal development of the majority of ciliary proteins displaying defects in forebrain development (Ftm, Ift172, Ttc21b, the hypomorphic allele of *Ift88*, Kif3a) [12], [13], [14], [15], [16], remains still undefined. Complete inactivation of *Ift88* causes absence of primary cilia both during embryonic development and in adult life [85], [86], whereas the role of Ift172 in ciliogenesis in the adult life is still undefined [87]. Interestingly, recent data suggest that the assembly of primary cilia is a critical event in the dendritic refinement and synaptic integration of adult-born neurons [88]. Our study suggests that conditional inactivation of *Ofd1* at birth does not affect formation of neuronal primary cilia during post-natal life. In fact, the comparison between male Ofd1-indKO (complete null) and heterozygous female embryos (mosaics for Ofd1) showed that Ofd1 is dispensable for ciliogenesis during post-natal life, but it is crucial during embryonic development. We hypothesize that the Ofd1 ciliary protein can play different roles in ciliogenesis depending on the developmental stage. Further studies using cell-type specific inducible *Cre-recombinases* at different time points might help elucidating the exact role of Ofd1 during development and at post-natal stages.

### Ofd1 mutants display actin disorganization

Several studies have revealed that actin remodelling is crucial for ciliogenesis [89] and that actin accumulates at the cell apex and it is required for basal body docking and subsequent axoneme assembly [66]. Remarkably, we observed actin disorganization and diminished apical enrichment of actin in severely affected *Ofd1^Δ^* ^4–5/+^ mutants, despite basal bodies being correctly orientated and docked. Thus, actin disorganization could be a consequence rather than a cause of defective ciliogenesis in *Ofd1^Δ^* ^4–5/+^ mutant females, suggesting that actin remodelling also occurs after basal body docking. Accordingly, a high-throughput functional screen recently identified modulators of ciliogenesis involved in actin dynamics [90]. Here, we demonstrate that cell adhesion and apico-basal cell polarity were affected in *Ofd1^Δ^* ^4–5/+^ mutants displaying a severe phenotype.

In summary, the current study extends our knowledge regarding the role of the basal body protein Ofd1 in forebrain development and may help explaining the neuropathological findings observed in patients bearing mutations in the OFD1 transcript and affected by OFD type I [18] and Joubert syndromes [25], [91]. Similarly to other ciliary proteins, Ofd1 is crucial for dorso-ventral patterning of the telencephalon. We demonstrate that ventralization of the telencephalon is due to altered Shh signalling associated with defective Gli3 processing. Moreover, the ultrastructural analysis reported here improves our understanding of Ofd1 function in ciliogenesis and demonstrates that Ofd1 is essential for ciliary axoneme elongation but is not required for basal body docking and maturation in the developing forebrain. We suggest that cytoskeletal rearrangements are most likely secondary to defective ciliogenesis and we report apico-basal cell polarity defects in *Ofd1* severe mutants. Finally, *Ofd1* might have a developmental stage-dependent role in primary cilia formation in the cortex.

## Materials and Methods

### Ethics statement

All animal experimentation was done under regulation of the Animal Care and Use Committee of the Cardarelli Hospital Naples, Italy to which our Institute (the Telethon Institute of Genetics and Medicine) refers to and authorized by the Italian Ministry of Health. The appropriate ethics committee specifically approved this study. According to Italian regulations and guidelines no permit number was issued.

### Mouse and genotyping

The generation of *Ofd1* knock-out mice and PCR genotyping were previously described [24]. Noon of the day of the vaginal plug was considered day 0.5 of gestation (E0.5). Embryos were stage-matched to controls by day count.

The CAGG-creER™ mice were obtained from Jackson laboratories and were generated by Dr. A. McMahon [63]. For induction of Cre activity at birth in the inducible model, tamoxifen administration was performed once at E18.5 in the pregnant mother *Ofd1* ^flox/flox^ crossed with CAGG-creER™ male. Tamoxifen (Sigma, St. Louis, MO) dissolved in corn oil (Sigma) was administered by intraperitoneal injection at a dose of 75 µg/g body weight. *Ofd1* ^flox/y^ and *Ofd1* ^flox/y^; CAGG-creER™ animals were analyzed 30 days post tamoxifen injection.

### Tissue preparation

Embryos at E12.5 and E18.5 were obtained by dissection in Dulbecco's phosphate-buffered saline (PBS). For *in situ* hybridization (ISH), embryos were fixed overnight in 4% paraformaldehyde in PBS pH 7.4 and subsequently cryoprotected in a gradient scale of sucrose (10%, 20% and 30%), embedded and frozen in Optimum Cutting Temperature compound (Tissue-Tek). Brains were serially sectioned at the cryostat (12 µm). For immunohistochemical analysis, they were fixed overnight with 4% paraformaldehyde in PBS pH 7.4, dehydrated, and embedded in paraffin wax. Brains were serially sectioned at the microtome (10 µm).

### Histology and immunohistochemistry

Nissl staining was performed on cryosections using standard procedures. For immunohistochemical studies, tissue sections were deparaffinized, rehydrated and processed. The following antibodies were used: polyclonal rabbit anti-Nkx2.1 (kind gift from R. Di Lauro, Stazione Zoologica Anton Dohrn, Naples), polyclonal rabbit anti-Tbr1 and anti-Tbr2 (1∶1000, kind gift from R. Hevner, Seattle Children's Research Institute, Seattle). Stained sections were visualized on an AxioPlan2 microscope and AxioCam CCD camera (Zeiss). Both histological and immunohistochemical staining were performed on three different embryos per genotype.

### In situ RNA hybridization (ISH)

The embryos for ISH on cryostat sections were fixed and sectioned as described previously [92]. We used the following probes: *Pax6* (kind gift from P. Gruss, Max Planck Institute of Biophysical Chemistry, Göttingen) and *Dlx2* (kind gift from J. Rubenstein, University of California, San Francisco). Partial complementary DNA (cDNA) of *Ofd1*, *Ngn2*, *Lhx2*, *Wnt8b*, *Mash1*, *Gsh2*, *Sfrp2*, *Dbx1* and *Gli3* genes were obtained by Reverse Transcription-PCR and then subcloned into TOPO cloning vector (Invitrogen). Digoxigenin-labelled RNA probes were prepared by *in vitro* transcription with the Digoxigenin RNA Labeling Kit (Roche) using T7 or Sp6 RNA polymerases. Sections were incubated overnight at 68°C in prehybridization buffer containing 200 ng/ml of digoxigenin-labeled RNA probe. Immunodetection of the hybridized probe was carried out using an anti-digoxigenin antibody (1∶2000, Roche). ISH images were visualized on an AxioPlan2 microscope and AxioCam CCD camera (Zeiss). All *in situ* RNA hybridizations were performed on three different embryos per genotype.

### Immunofluorescence

For immunofluorescence analysis, brain cryosections were blocked for 30 minutes with 1% BSA, 2% goat serum in PBS/0.3% TritonX-100 and then incubated with primary antibody overnight at 4°C. The following antibodies were used: rabbit anti-Caspase 3 (1/500, BD Pharmigen), rabbit anti-Adenylyl cyclase type III (clone C-20, 1/500, Santa Cruz Biotechnology), mouse monoclonal anti-γ tubulin (1/2000, Sigma), mouse monoclonal anti-β catenin (1/1000, Santa Cruz Biotechnology), rabbit polyclonal anti-ZO-1 (1/400, Zymed), rabbit polyclonal anti-PAR3 antibody (1/500, Upstate), mouse monoclonal anti-Ki67 (1/200, BD Pharmingen). For Ki67 experiment, sections were heated at 95°C in citrate buffer pH 6.0 for 7 minutes before incubation with primary antibody. The sections were then washed with PBS and incubated with secondary antibody. For F-actin staining we used Phalloidin-TRITC conjugated (1/1000, Sigma) diluted in PBS pH 7.4. Nuclei were counterstained with 4′,6-diamidino-2-phenylindole (DAPI) and stained sections were mounted with Vectashield (Vector Laboratories). Microscopy was performed with a Zeiss Axioplan 2 microscope and with Leica TCS SP2 AOBS confocal microscope with a 63× Neofluor Pan-Apo 1.3 nm oil objective. Every immunofluorescence was performed on three different embryos per genotype. For analysis at P30 we used three different brains per genotype.

### Western blotting

For western blotting analysis of Gli3, whole-cell lysates were prepared from brains of E12.5 wild-type and *Ofd1* ^Δ4–5/+^ mutant females using RIPA buffer [10 mM Na-phosphate ph 7.2, 150 mM NaCl, 2 mM EDTA, 1% NP-40, 1% Na-deoxycholate, 0.1% SDS, protease inhibitors cocktail (Roche)]. Heads from three different embryos per genotype were disrupted using a mini-pestle (Bio-Optica) prior to protein extraction. Equal amounts of protein were loaded onto 7% SDS-PAGE gels and western blotting was performed as described [93]. The membranes for the Western were probed using a goat polyclonal anti-Gli3 (1/250, R&D Systems), then stripped and probed with a mouse monoclonal anti-β-tubulin (1/3000) as loading control. Densitometry was used to compare protein levels between the full-length activator and truncated repressor forms of Gli3 (ImageJ 1.37v software available at http://rsb.info.nih.gov/ij/).

### Real-Time PCR analysis

Whole brain from animals were dissected and washed with ice-cold PBS. They were mixed and homogenized in Trizol reagent (Life Technologies). Total RNA was then purified on RNeasy columns (Qiagen). cDNA synthesis was performed according to manufacturer's instructions (SuperScript kit; Invitrogen). For Real Time (RT)-PCR, cDNA and primers were mixed with SYBR-green RT-PCR Master Mix (Roche Applied Science) and then assayed in a LightCycler®480 RT-PCR detection system (Roche Applied Science) as directed by the manufacturer. The relative level of each mRNA was calculated using the standard curve method and normalized to the corresponding *Hprt* mRNA levels. Five independent RNA samples were used for each group (e.g., one group was *Ofd1^Δ4–5/+^* mice, severe phenotype) and triplicate reactions of each sample were used to derive the normalized expression level for each gene. The average normalized expression levels were used to determine the average expression level within a group, and for statistical comparisons between groups (thus, N = 5 for each group). ANOVA and Student t-tests were performed to measure variations in gene expression between groups.

### Oligonucleotides

The primers for each gene analyzed were designed with Primer3 software: Ofd1F: 5′-TGGCAGACCACTTACAAAGATG-3′; Ofd1R: 5′- AGACTGGATGAGGGGTTAATC-3′; ShhF: 5′-CCCAAAAAGCTGACCCCTTTA-3′; ShhR: 5′-TTCCCTTCATATCTGCCGCT-3′; Gli1F: 5′-TGCAGTAAAGCCTTCAGCAATG-3′; Gli1R: 5′-TTTTCGCAGCGAGCTAGGAT-3′; Ptch1F: 5′-CCACGACAAAGCCGACTACAT-3′; Ptch1R: 5′-GCTGCAGATGGTCCTTACTTTTC-3′; Gli3F: 5′-GCTGGCTTGATTGTTCACGA-3′; Gli3R: 5′-GGCTTTTGTGCAACCTTCAAA-3′; SmoF: 5′-CAGTTCCAAACATGGCAAACAG-3′; SmoR: 5′-TGCTATGTGAGGCCAATGTGA-3′. The primers used for the analysis of genomic DNA are the followings: Ofd1F: 5′-CATTCCTGTTAGTATTTGGAGG-3′; Ofd1R: 5′-GTGTTAGGAGGGTATGAACATG-3′; GapdhF: 5′-TCTTCTGGGTGGCAGTGAT-3′; GapdhR: 5′-TGCACCACCAACTGCTTAGC-3′.

### Scanning electron microscopy (SEM)

Neocortex from *Ofd1* ^+/+^ wild-type and *Ofd1* ^Δ4–5/+^ mild and severe embryos at E12.5 were isolated in 1.5% glutaraldehyde, 0.067 M Cacodylate buffer pH 7.4 plus 1% sucrose and after were fixed in the same fixative for 4 h at 4°C. Then they were rinsed in 0.134 M Cacodylate buffer pH 7.4 overnight. Post fixation was based on 1% osmium tetroxide solution (Fluka) in 0.067 M Cacodylate buffer pH 7.4 plus 1% sucrose cooled on ice for 1 h. After several rinses in 0.134 M Cacodylate buffer pH 7.4, the specimens were subjected to serial dehydration followed by critical point drying. The samples were mounted on aluminium stubs and sputter coated with gold. The processed specimens in the area of the ventricular zone were investigated and photographed using a JEOL 6700F SEM operated at 5 kV and at 8.3 or 3.1 mm working distance. SEM images were collected digitally. SEM analysis was performed on three different embryos per genotype.

### Transmission electron microscopy (TEM)

Neocortex from *Ofd1* ^+/+^ wild-type and severe *Ofd1* ^Δ4–5/+^ embryos at E12.5 were isolated in 1.5% glutaraldehyde, 0.067 M Cacodylate buffer pH 7.4 plus 1% sucrose and after they were fixed in the same fixative for 4 h at 4°C. Then, they were rinsed in 0.134 M Cacodylate buffer pH 7.4 overnight. Post fixation was based on 1% osmium tetroxide solution (Fluka) in 0.067 M Cacodylate buffer pH 7.4 plus 1% sucrose cooled on ice for 1 h. After several rinses in 0.134 M Cacodylate buffer pH 7.4, specimens were dehydrated with ethanol and then, with propylene oxide and embedded in Epon 812 resin (Fluka). The blocks were cut using a Super Nova Leica Ultratome. Semithin sections at 2 µm thickness, were studied with a light microscope (Polivar Reichert-Jung) after staining with 1% toluidine blue (Carlo Erba). Ultrathin sections (80 nm) were stained with 2% uranyl acetate (Electron Microscopy Sciences) for 10 min at room temperature and 2.66% lead citrate (Electron Microscopy Sciences) for 3 min at room temperature. Grids were examined by using a JEM-1011 Jeol transmission electron microscope operating at 100 kV. TEM analysis was performed on three different embryos per genotype.

### Statistics

All error bars represent one standard deviation. For immunofluorescence quantifications, at least 200 cells were counted on each duplicate coverslips in at least two separate experiments. Student's unpaired t test was used to determine statistical significance with a p value of less than 0.05.
